# Socio-cultural and economic barriers, and facilitators influencing men’s involvement in antenatal care including HIV testing: a qualitative study from urban Blantyre, Malawi

**DOI:** 10.1186/s12889-020-10112-w

**Published:** 2021-01-06

**Authors:** Doreen Sakala, Moses K. Kumwenda, Donaldson F. Conserve, Bassey Ebenso, Augustine T. Choko

**Affiliations:** 1 Public Health Group, Malawi-Liverpool-Wellcome Clinical Research Programme (MLW), Blantyre, Malawi; 2 Helse Nord TB Initiative, College of Medicine, Blantyre, Malawi; 3 Department of Health Promotion, Education, and Behavior, University of South Carolina, Arnold School of Public Health, Columbia, SC USA; 4 University of Leeds, Institute of Health Sciences, Leeds, UK

**Keywords:** Antenatal care, HIV, HIV testing, Male involvement, Malawi, Sub-Saharan Africa

## Abstract

**Background:**

Male partner involvement in antenatal care (ANC) is associated with positive maternal and neonatal outcomes. However, only a handful of men attend ANC with their partners. This study aimed to understand the underlying barriers and facilitators influencing men’s ANC attendance including HIV testing in Blantyre, Malawi.

**Methods:**

Data were collected during a formative qualitative study of a cluster-randomised trial. Six focus group discussions (FGDs) with 42 men and women and 20 in-depth interviews (IDIs) were conducted at three primary health centres in urban Blantyre, Malawi. FGD participants were purposively sampled with IDI participants subsequently sampled after FGD participation. Thematic analysis was used to analyse the data.

**Results:**

The economic requirement to provide for their families exerted pressure on men and often negatively affected their decision to attend ANC together with their pregnant partners despite obvious benefits. Peer pressure and the fear to be seen by peers queueing for services at ANC, an environment traditionally viewed as a space for women and children made men feel treated as trespassers and with some level of hostility rendering them feeling emasculated when they attend ANC. Health system problems associated with overall organization of the ANC services, which favours women created resistance among men to be involved. An association between ANC and HIV testing services discouraged men from attending ANC because of their fear of testing HIV-positive in the presence of their partners. The availability of a male friendly clinic offering a private, quick, supportive/sensitive and flexible service was considered to be an important incentive that would facilitate men’s ANC attendance. Men described compensation to cover transport and opportunity cost for attending the clinic as a motivator to attending ANC services and accepting an HIV test.

**Conclusion:**

Peer and economic influences were the most influential barriers of men attending ANC and testing for HIV with their pregnant partners. Addressing these socio-economic barriers and having a male friendly clinic are promising interventions to promote male ANC attendance in this setting.

**Supplementary Information:**

The online version contains supplementary material available at 10.1186/s12889-020-10112-w.

## Background

Antenatal care (ANC) presents an opportunity for health screening to both the female and male expectant partners [1]. For example, up to 94% of women attending ANC accept to test for HIV globally [2]. However, this opportunity remains underutilized to reach male partners of ANC attendees in contexts with high HIV prevalence such as sub-Saharan Africa (SSA) [3]. This is of concern considering that men in general are less likely to test for HIV [4] and consequently have much higher death rates because they start HIV treatment late compared to women in SSA [5]. In addition, SSA accounts for 70% of the global HIV burden including illness and deaths [6], making it essential for men and women in SSA to be routinely screened for HIV.

Recent studies have demonstrated that ANC may hold the key to reaching male partners in SSA with HIV testing services [7–10]. In addition, male involvement which involves actively engaging the male partner during pregnancy in ANC services is associated with benefits to both partners and the unborn baby [10, 11]. For example, a cohort study in Kenya reported decreased infant HIV infection and lower neonatal deaths among women with male involvement [11]. Men who attend ANC services with their female partners are more likely to test for HIV [12, 13].

The literature identifies dominant barriers around socio-economic factors such as level of income, space constraints at ANC, men’s beliefs that ANC is for women [14–17]. Barriers to male involvement in ANC during pregnancy may operate at different levels including individual, couple, community, and health system [17] and their impact on men’s behaviour may be context specific [14, 17].

The Malawi antenatal care programme follows the WHO/UNICEF report of 2003 which recommends at least four clinic visits for pregnant women according to the three trimesters [18]. The initial visit which normally occurs during the first trimester may play a key role in encouraging any support from the male partner during pregnancy (*aka* male involvement) [19]. Up to 95% of pregnant women in Malawi attend antenatal care with the majority of attendees accepting and being tested for HIV [20]. However, male involvement is very low: only 19% of male partners included in a recent trial achieved male involvement in Malawi [21]. Such low male involvement leads to underutilization of HIV services among men during pregnancy. In Malawi generally, women are more likely than men to have ever been tested for HIV (83% versus 70%) [20] with a similar gap in treatment initiation [22]. In particular, HIV testing uptake remains lower among male partners compared to their pregnant female partners. While up to 85% of pregnant women were tested for HIV in a 2010 study [23], just over 40% of women reported that their male partners had never tested for HIV in a recent (2019) trial [7].

This study investigated barriers and facilitators influencing men’s decision-making regarding ANC attendance in Blantyre, Malawi. Results presented in this paper may contribute to the literature that informs the direction towards identifying effective interventions for increasing male involvement in Malawi and similar contexts. This study utilized social identity theory which posits that individuals place greater importance and value in belonging to a group to the extent that they may alter their own behaviour, attitude and practices to conform to the group’s dictates [24]. We sought to apply this theory with respect to men’s perspectives regarding male involvement in ANC.

## Methods

### Study design, participants and data collection approaches

This was an analysis of primary data from a formative qualitative study of a multi-arm, multi-stage cluster randomised trial from a study called Partner Assisted Self-testing and Linkage (PASTAL ISRCTN18421340) [25]. The study was conducted between 8 August 2016 and 30 June 2017 at three primary health centres (PHCs) of Zingwangwa, Ndirande and Bangwe located in urban Blantyre, Malawi. Of the three PHCs, Zingwangwa had the highest ANC throughput and offered ANC to first time attendees on Friday only, whereas Bangwe and Ndirande offered it on Monday only. Pregnant women attending ANC for the first time as well as male partners of ANC attending women, not necessarily as couples participated in focus group discussions (FGDs) and in in-depth interviews (IDIs).

Qualitative data were collected through six focus group discussions (FGDs) involving a total of 42 ANC attendees and male partners, and 20 in-depth interviews (IDIs) conducted with ANC attendees and male partners who had participated in the FGDs. FGDs were used to enable researchers elicit collective views that represented community perceptions around the barriers and facilitators of male partners’ attendance to ANC through group discussions. The follow-on IDIs built on the FGDs and solicited more intimate-personal views using different although somewhat similar set of questions. This approach strengthened the quality of the data collected because data from FGDs provided a global view whereas data from IDIs provided a much finer view and potentially eliminated bias from group level influences in responses. Thus, combining FGDs and IDIs allowed triangulation of data through constant comparison of the results.

### Sampling

**Fig. 1:**
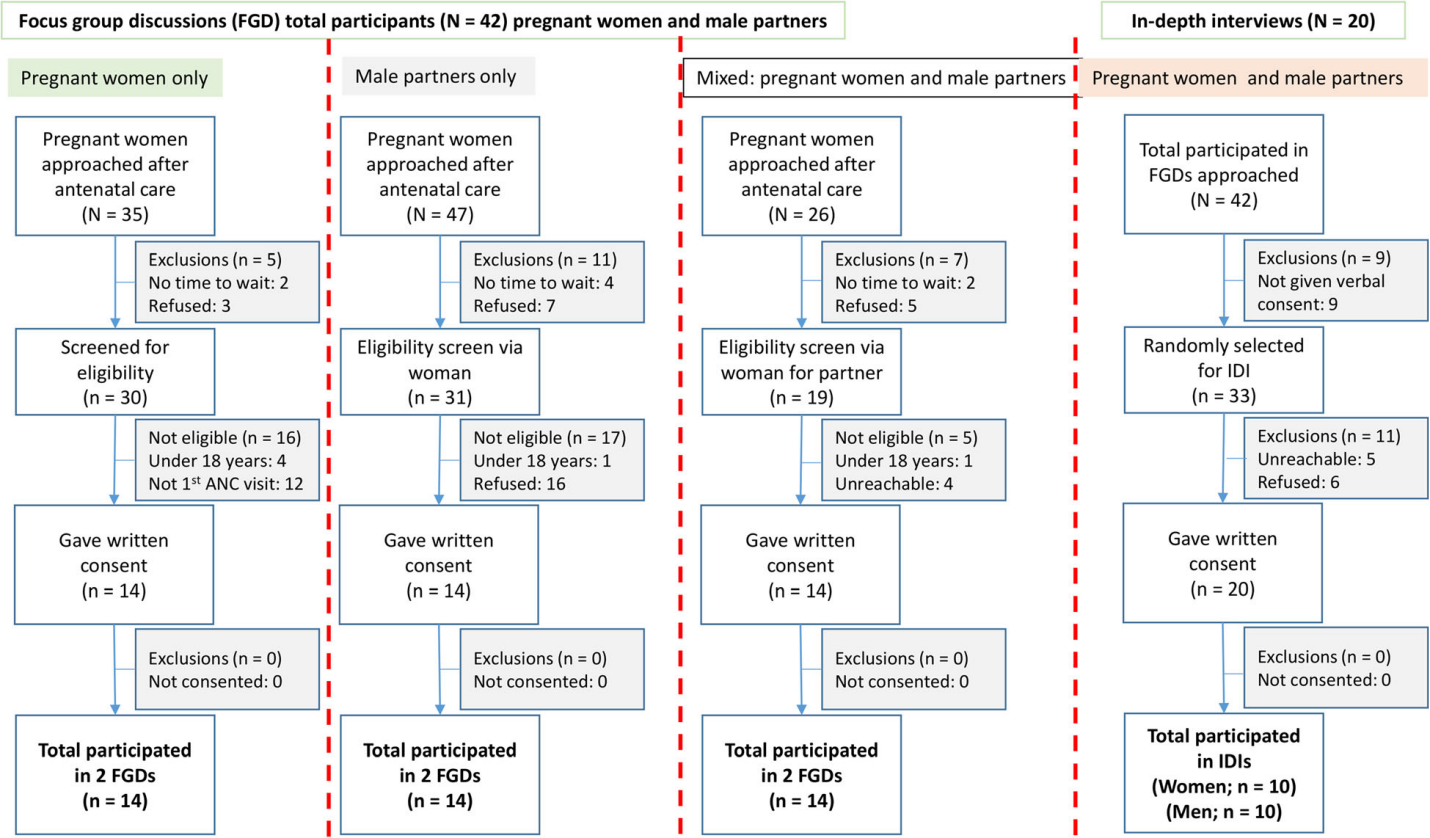
Description of recruitment for focus group discussions and in-depth interviews

Purposive sampling was employed with the aim of recruiting ANC attending pregnant women to participate in the FGDs and as a way of reaching out to their partners. A research staff member worked with the nurse on duty to identify and approach pregnant women for inclusion in the study. To be eligible, women had to be 18 years and above, and visiting ANC for the first time. Sampling was implemented during pregnant women’s routine visit to the ANC at the selected PHC (Fig. 1). While ANC attendees waited in a group at a waiting bay to access ANC services, the study team utilised the routine early morning health education talks to introduce themselves and the study by providing oral information on its purpose and the importance of their participation. Recruitment aimed to have between 8 to 12 participants for the FGDs and from each completed FGD, 4 participants for the IDIs. After accessing the ANC services, women were then approached individually and requested to voluntarily participate in the study. Women who showed interest were requested to provide an informed consent and recruited for the FGD which took place immediately after receiving the ANC service.

Women who participated in FGDs were given a letter inviting their male partners to participate in the study. The partner’s phone number was obtained from the women during the group discussion. Male partners were contacted via a phone call after 5 o’clock in the evening as this was suggested by most women to be the most convenient time as they would have clocked off from work or business. During the phone call, eligibility screen was administered: aged 18 and above and being available in the catchment area in the next 28 days. Male partners who verbally consented were then informed about participating in FGD and IDIs, venue and time for the interviews.

All male partners and women who participated in FGDs were eligible to participate in IDIs. Therefore, all FGD participants were approached and asked for oral consent to participate in IDIs. A random selection of the final IDI participants was drawn from all FGD participants who orally consented to participate in IDIs. Written consent was obtained from all participants before completing an IDI. All FGDs and IDIs were conducted either at the health centre for women or at a convenient place in the community (e.g. school) as preferred by participants. FGDs and IDIs were done in the local language (Chichewa) and were led by social scientists (DS and MK) who are both fluent in the local language of use. Participants received ~US$5 for their time.

### Data collection

**Fig. 2:**
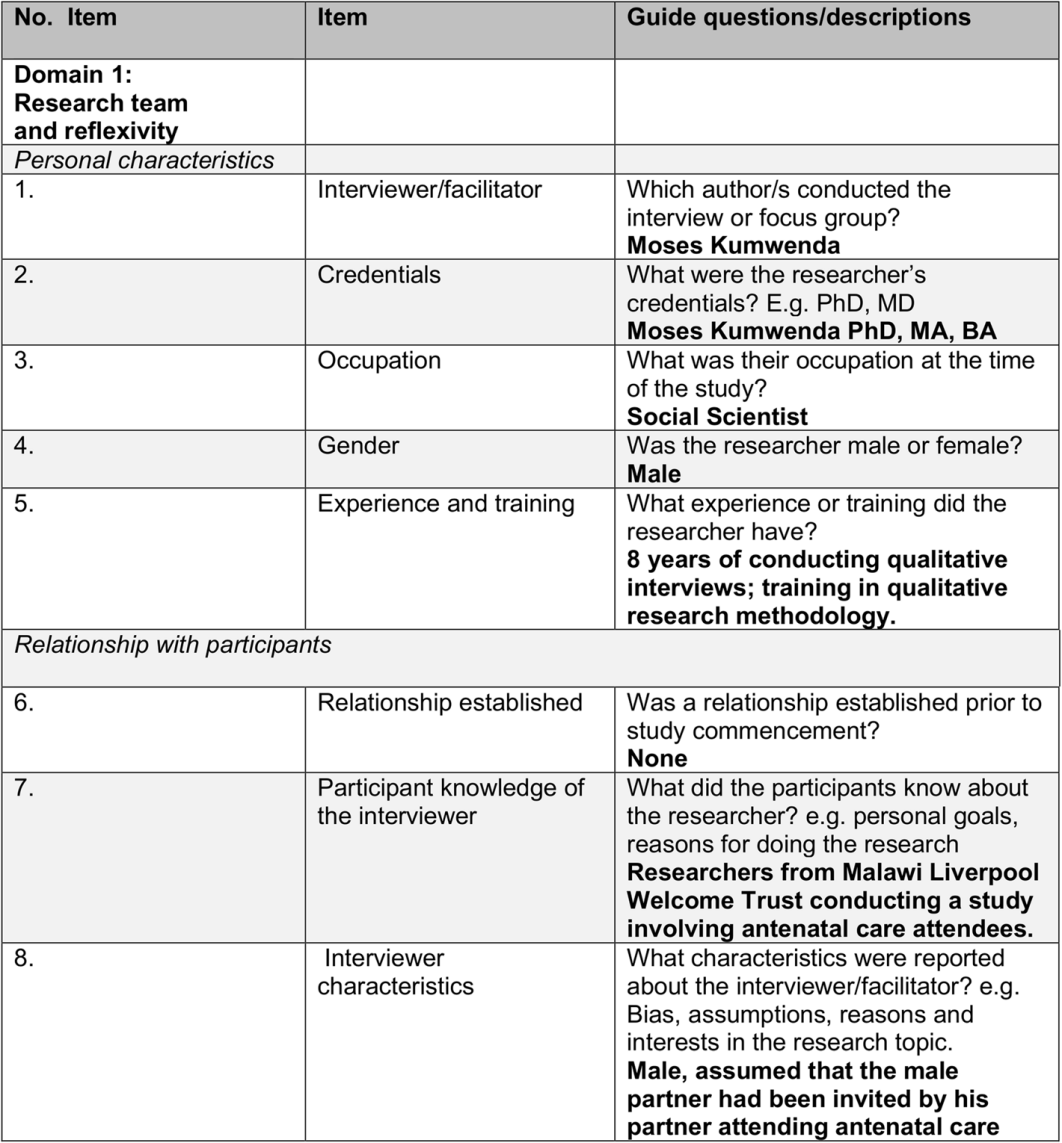
Consolidated criteria for reporting qualitative research (COREQ) checklist

Data were mainly collected by two researchers, a senior social scientist (MK) with a PhD led FGDs while a research assistant (DS) with a BSc took the field notes during the FGD’s and led the IDIs (Fig. 2). Research participants had no relationship with the researchers although women had interacted with the researchers before engaging the male participants. A semi-structured question guide was used during the FGDs (Additional file 1) and the IDIs (Additional file 2) with embedded and flexible probes to prompt additional discussion and correct course of discussion. Both the FGD and the IDI guides focused on perceptions of the ANC service, perceptions of HIV testing by male partners during ANC, and perceptions of HIV self-test kits delivered to male partners at home by a pregnant woman. Notable differences between the FDG and the IDI guide were the individualized nature of the IDI questions and probes around whether or not the individual would accept to distribute (pregnant woman) or receive (male partner) HIV self-test kits provided during ANC.

All FGDs and IDIs were recorded with digital recorders and the recorded qualitative data were transferred onto a computer, before translation and transcription. The FGD’s were in-between 1 h to 1 h 30 min, whilst the IDI’s lasted a maximum of 30 min. The FGD and IDI participants also completed a short questionnaire to collect their demographic data and these were analysed quantitatively (see Fig. 5 in results section). Apart from the participants and interviewers, there was no one present in the data collection rooms.

### Data analysis

**Fig. 3:**
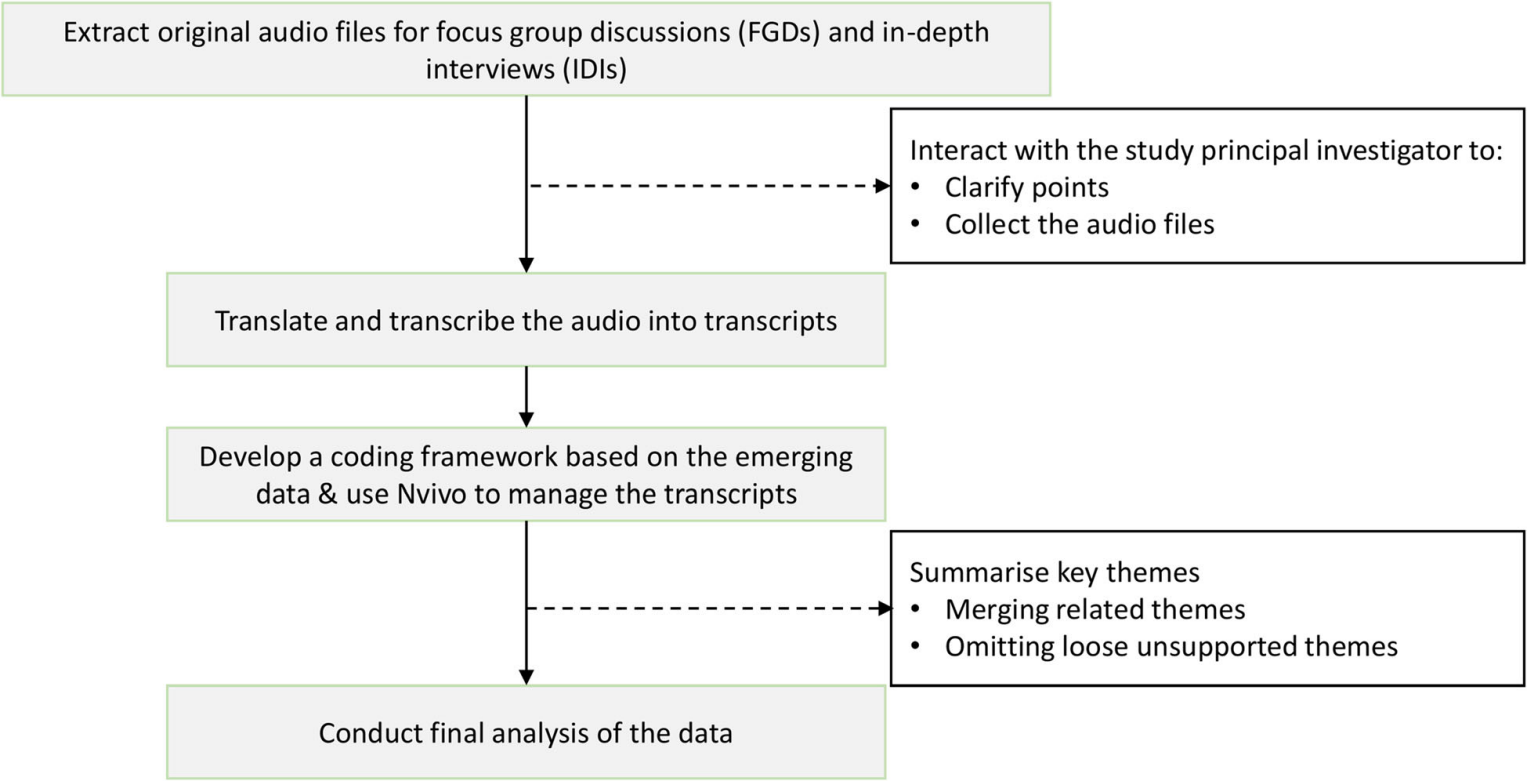
Summary of data analysis process

Nvivo version 10 was used for data organization and analysis following translation and transcription of the six FGDs and 20 IDIs. A coding framework was developed separately for FGDs and IDIs based on the question guides. Thematic analysis was used to analyse qualitative data [26]. Data analysts (MK, DS, AC) familiarised with the data by reading and re-reading transcripts to look for implied meanings and try to see the data in context [26]. Inductive and deductive coding was done by developing an initial coding framework based on the research objectives and also reading transcripts multiple times while attaching labels to data on the basis of meanings that the researcher discerned in the data [26]. Data were conceptualised by constant comparison of points raised by different people within and across FGDs and IDIs. Themes were developed by grouping several categories/codes that represented a unified subject/topic (Fig. 3). Data are presented as a descriptive narrative with quotes used to support each emerging theme.

**Fig. 4:**
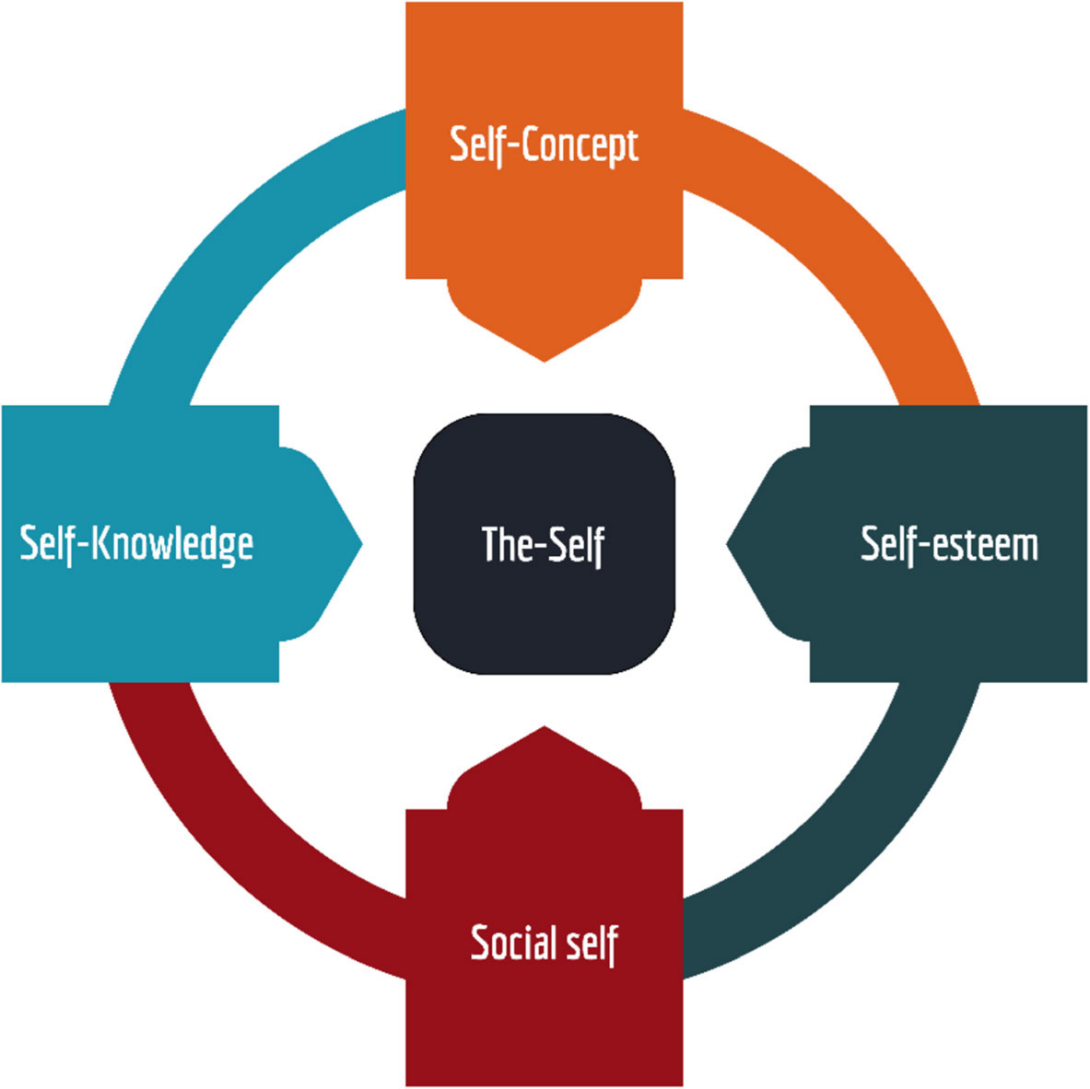
Components of the theory one’s self. *Source:* Modified from Social Identity theory Tajfel (1971) British Journal of Social and Clinical Psychology

The overall analysis was guided by the social identity theory which was introduced by social psychologists Henri Tajfel and John Turner in the 1970’s [24]. The theory is based on the idea that people categorise themselves based on the social groups to which they belong (Fig. 4). The theory has three processes namely; social categorization, whereby people are defined based on social categories rather than their individual categorization. The second process is social identification where people tend to behave in the way that they believe their group should behave. The third is social comparison where people compare themselves to another group in terms of having a higher or lower social standing. A number of factors were at play in the male decision-making process to go to ANC and test for HIV and these were critiqued in light of the social identify theory in this study. Most factors involved men categorising themselves in different groups affecting their decision to attend ANC and have an HIV test.

## Results

### Participant characteristics

**Fig. 5:**
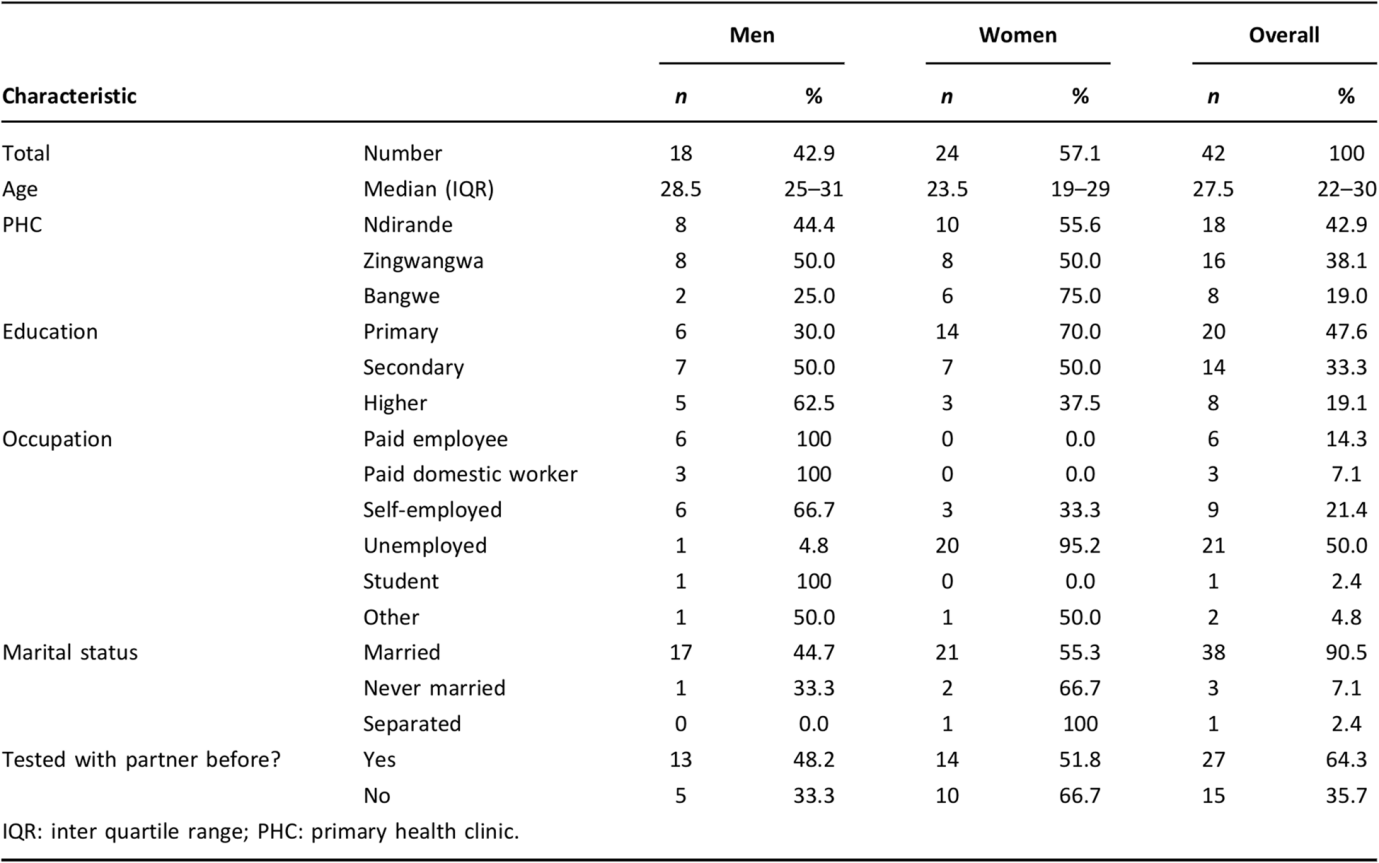
Characteristics of all study participants by sex*.* Reproduced with permission from Choko et al. JIAS 2017

Overall, the study had 42 participants, 18 men and 24 women (Fig. 1). Participation was notably higher among women 57% (24/42) (Fig. 5) than men who were eligible partly because male partners were reached via the women (Fig. 1).. Men were older with median age of 28.5 years (interquartile range [IQR]: 25–31) while median age for women was 23.5 (IQR: 19–29). There was generally equal representation of female participation from the three clinics although in Bangwe there were fewer men: 2 vs 8 each in Ndirande and Zingwangwa. More women than men reported lower level of formal education with 70% of the women having completed primary school only compared to only 30% of the male partners. All six participants who said they were paid employees were men with none of the 24 women reporting being in any form of paid employment. Overall, 90.5% (38/42) of the participants reported that they were married. Of the participants who did not previously test for HIV, 33.3% (5/15) were men and 66.7% (10/15) were women. Social desirability bias may explain this finding as women are consistently found to have higher HIV testing percentage in the literature [4].

## Results

Factors influencing men’s decision-making process to attend or not to attend ANC with their pregnant partners and testing for HIV have been organised into two broad themes namely: barriers and facilitators to male partner involvement. Five sub-themes emerged under the barriers to male partners of ANC attending women, while four sub-themes emerge as facilitators to male involvement.

### Barriers of male partner’s decision to attend ANC and test for HIV

The five key factors that influenced male partner’s decision not to attend ANC with a pregnant partner and test for HIV in ANC clinics were community norms and beliefs, peer influence, risk of attending ANC and opportunity cost associated with attending ANC services. These are briefly discussed next.

### Community norms and beliefs

Views of the societal norms and long held traditional beliefs regarding the roles and responsibilities of women and men seemed to play a major role in the decision making regarding male partner ANC attendance and testing for HIV at ANC clinic during a scheduled clinic visit for a female partner. ANC was traditionally considered to be spaces restricted to women and therefore not suitable for male partners because the focus of the services in these clinics was mainly directed to benefit pregnant women.

“Some people (in the community) say such men have nothing to do that is why they go there (to antenatal care), explaining that if they had some work to do they would not be wasting time going there”. Ndirande PHC-Female FGD.

The quote demonstrates how community norms fuel the perception that a man should not attend ANC together with his pregnant partner. It also highlights how men who attend such clinics are perceived to have nothing productive to do.

### Peer influence

Male partner’s peers and social networks seemed to influence their decision to either attend or ignore attending ANC even at the request of their pregnant partners. Male partners frequently reported that their peers would not consider attending ANC with their partners. Male partner’s ability to conform to the ideals and values of their social networks was visibly determined by how they conducted themselves within the network. The following quote demonstrate how opinions of peers could alter male partner’s perspectives and eventually the decision about attending ANC and test for HIV:

“My friends were just laughing at me, for example two days ago before I came here, I told them my wife wants to start going to ANC. I want to go with her to see how the process is like. My friends said, ‘you are stupid because ANC is for women only, not for men’”. Ndirande PHC-Male FGD.

The above quote demonstrates how peers ridicule and demean other men simply for attending ANC with their partner and how a circle of friendships influences men’s attitude and behaviour towards attending ANC. It also vividly illustrates real life experiences of men emasculated or perceived as not being a man enough just because they chose to attend ANC clinic. One participant described how opinions from friends about ANC are taken into consideration during decision-making processes to eventually deter male partners who may have not been totally against attending ANC initially:

“As someone already said, most men talk about discouraging things. So, when you pay so much attention to them, you tend to follow your friends – ‘ … the way my friends talked, I can’t be going there with my wife.’ So, when you take those things seriously, and you were also doubting in the first place then that’s it”. Zingwangwa FGD Male.

The influence of male network members on male partner’s decision to attend ANC was also described by female participants, who narrated that women often find it challenging to convince their partner to attend ANC. Female participants were aware that most male partners are discouraged from attending ANC by their close friends as illustrated by the following quote:

“It’s mostly men themselves who discourage each other. For us women, we may be encouraging the men to be coming with us to antenatal clinic but you find that their friends are the ones who make them lazy by telling them ‘how can you let a woman lie to you? How can you go to the hospital with a woman? Don’t accept that, you are stupid to do that.’ With such discouragement if the man had thoughts of going to the hospital, he becomes lazy as well because of what his friends said”. Zingwangwa FGD Female.

The quote demonstrates how male partners, influenced by their peers, attempt to enact their masculine roles by avoiding being associated with ANC which is traditionally considered to be associated with women. Accepting to attend ANC clinic with a female partner would implicitly be relinquishing one’s masculine power and control in submission to demands of a female partner.

Perceived social risks associated with male partner's ANC attendance in a cultural context of reward and sanctions, male partners anticipated that they would encounter certain negative consequences from their social networks because they opted to attend ANC clinic with their pregnant partner and this would damagingly affect their social image and position. The perceived negative reactions from their social network that male partners expected included weakening their male friendship bonds, being perceived as fearing and subordinate to one’s partner, and being disrespected by their male friends as captured in this quote:

“Even the relationship or friendship one had with his friends gets affected. When you go to ANC, it’s more like you will just be chatting with your wife only rather than chatting on our own as men.” Zingwangwa FGD Male.

Thus, men were afraid to lose their social identity in the community but also the social capital that they have built by merely choosing to visit ANC clinic with a pregnant partner. Another participant described the perception that men who attend ANC are either afraid of their partner or that the woman cast a magic spell on the man through witchcraft to coerce him to attend ANC which he would have otherwise not accepted in his normal self.

“Others say ‘the man is stupid and he is scared of his wife. That’s why he has gone to ANC for testing.’ They say ‘the woman has forced him to go get tested’ while others say that ‘he is scared of his wife or maybe the wife used traditional medicine’.” Ndirande Mixed FGD Male.

In the quote above, a male partner’s choice to attend ANC is interpreted as either being foolish or as a sign of subordination to the demands of a wife or simply lack of power in the relationship. Furthermore, supernatural forces are sometimes used to explain this kind of behaviour which is perceived as being unnatural for the male gender identity. The risk of losing respect in one’s network is a concern for some male partners who attend ANC, with one participant reporting that he witnessed men disrespecting male partners of pregnant women who had attended ANC:

“I have been seeing other men looking down upon or saying rude things to other men who have escorted their wives to the hospital at ANC. They say things like ‘he is stupid’ or maybe ‘I cannot participate in escorting my loved one to ANC’, or maybe ‘I am busy’.” Bangwe IDI, Male.

### Opportunity cost/time conflict/food insecurity

The amount of time required for male partners of pregnant women to be at ANC clinic was a challenge as most male partners who felt that visiting a clinic would keep them away from potential employment or economic opportunities that could help them provide for their families. This concern was amplified for poor men who were experiencing food insecurity and living in a precarious economic environment. Thus, the choice to go to an ANC clinic with a female partner conflicted with the male gender role to provide for the family as illustrated in this quote:

“It is true that many people say that a man who goes to ANC has nothing to keep him busy with. They say ‘if both a man and a woman go to ANC, who will go look for food? Will the family eat ANC?’” Zingwangwa IDI Male.

The quote above suggests seemingly clear food insecurity which often places men at the forefront of fending for the household. It also depicts stigmatisation associated with failure to provide for the family by men. For an employed male partner, their decision to attend ANC clinic with their pregnant partner was further compromised where the employer failed to allow them to leave their workplaces. Male study participants mentioned that it was difficult for employed men to be excused from work to attend ANC with their partner:

“I work at someone’s house and I have come here. So, another day comes I should tell them I am going to ANC again, they will say are you the one pregnant or what? You were supposed to go the first day of registration only, so most times you try to protect your job” Zingwangwa FGD Male.

The quote above clearly demonstrates that men may not have sufficient grounds to justify attending ANC clinics because biologically, they do not carry the pregnancy. However, there is a recognition by employers that men can attend the first ANC visit only.

### Fear to test for HIV

Almost all participants mentioned that because of stigma, male partners of ANC attending women were scared to test for HIV publicly at ANC. It was frequently mentioned that male partners would rather go for HIV testing by themselves first to see the outcome alone before going for the second time with their partners when they are confident about the test outcome. The quote below illustrates the need for male partners to have a separate initial HIV test before agreeing to attend ANC with their partner.

“It’s better to go behind her back or maybe go to Banja Lamtsogolo (HIV testing centre) or health centre to get tested and see the outcome by yourself first.” Ndirande PHC-Male FGD.

It seems that fear of learning one’s HIV sero-status for the first-time with a sexual partner was intimidating to most male partners especially if the test results came out HIV-positive. Male partners were also worried about the negative consequences associated with taking an HIV test together with a partner. Importantly, the fear of learning about HIV-discordant results together was perceived to lead to marriage breakup and inadvertently revealing infidelity especially of the male partner:

“The part that I feel like is tough is if my wife or let’s just say when two people want to get tested, the first thought is if one is found negative the other positive then that’s the end of the marriage.” Ndirande PHC-Male FGD.

The quote above highlights the lack of information on the part of the general population about managing HIV discordant couples and the role of treatment as prevention. As such, a default response to HIV sero-discordant result is to think about the incompatibility of sexual partners based on their divergent HIV serostatus resulting into marriage breakups.

### Facilitators of men’s decision to attend ANC

Several factors were identified to encourage male partners to decide to attend ANC with their female partners. These included an arrangement that would give priority to couples during consultations, health education targeting male partners, the benefit of couples testing and availability of a male friendly space within the ANC clinic.

### Priority consultation for couples

Study participants recognized that male partners would be encouraged to attend ANC where couples are given priority when undergoing formalities at ANC clinic and if they understood that their attendance at the clinic would mean that their female partner would receive prompt medical attention than the rest of women who present without a male partner. A quote below illustrates this:

“The ones [pregnant women] who come with their male partners are the first to be assisted so that the man should go back in good time to continue with his work. They both leave early that’s the good part of it.” Ndirande IDI, Female.

In the quote above, a female participant demonstrates that male partner’s knowledge that they would spend less time at a facility might encourage them to accept attending ANC with their female partner. However, it was not clear from the data whether the ANC clinics currently provided preferential treatment to couples and whether male partners are aware of this provision.

### Health education for the benefit of male partners

Another important factor that facilitated male involvement in ANC was a prospect of learning new things through the regular health talks at facilities before service provision starts. With a male partner in attendance, both partners get to hear about the health of the woman and the baby.

*“I have learnt a lot here, they educated us on the right types of food to eat, the right sides to sleep and how much exercise and work the woman can do which is very helpful because I was not aware of all this information.”* Ndirande PHC-Male FGD.

In the quote above, a man expresses excitement for learning new things about how to manage the gestation period to ensure smooth delivery of the baby.

### Value of testing together

It was also highlighted by participants that testing together for HIV helps them to know each other’s status hence cementing their relationship. In general, women saw greater value in testing together while male partners seemed to view testing together as a concern.

*“It’s good because you get to know each other’s status and since you are also counselled together, you are able to remind each other on what you were told to do so it’s helpful*.” Ndirande PHC-Female IDI.

*“With the fear of testing together as expressed by FGD participant number 1, actually the main fear* [with testing together] *is that maybe my* [HIV positive] *status will be known by everyone.”* Zingwangwa Male FGD.

### Male friendly clinics

Most participants said that having a male friendly clinic within ANC would allow male partners of ANC attending pregnant women to feel comfortable to visit a space that is traditionally associated with women and children:

*“Having a male friendly clinic can help if it can be aside* [away from the main clinic]*. Most men would go since they won’t have to be shy anymore knowing they will not have to sit amongst women. And if I am found* [HIV] *positive everything will be discussed right there.”* Zingwangwa IDI female.

The quote above suggests that the current arrangement of ANC services is not conducive to the male partners who feel outnumbered and uncomfortable to sit among women. However, having a male friendly clinic would provide a safe sanctuary for male partners.

## Discussion

The main findings from this analysis vividly demonstrated that peer pressure [27] and opportunity cost of attending ANC instead of being at work [28] exerted significant influences on male partners and played a major role in their decision to engage or attend antenatal care along with their pregnant partners. It was found that men as peers perceived themselves as a strong group hence did not see the need of listening to their female partner. Social interaction also influenced male partners of ANC attending pregnant women as they would rather chat with fellow men and not seen as always with a female partner. Concepts from the *social identity model* appear to explain in part the dynamics reported in this study. “Social identity is the portion of an individual’s *self-concept* derived from perceived membership in a *relevant* social group [29]”. In particular, individuals belonging to a particular group are expected to “behave” and conform to group norms and expectations. It was observed that narratives from the male partners of ANC attending pregnant women themselves appeared to support the idea that men often value the opinion of their peers as men more than their household members. Men’s view that they would not want to disappoint their friends or feel embarrassed in their company by attending ANC with their pregnant partner supported the notion of the “self” and in particular the self-construct (Fig. 3). Of note, study participants mentioned that the society and sometimes the health system perceived or expected male partners of ANC attending pregnant women not to attend ANC as it was a woman’s space [10, 16]. Although this can be construed as a cultural belief, it may also be a form of social identity ascribed to men as a group.

Economic influences from different dimensions were a critical aspect for male partners to consider before deciding to attend ANC with their pregnant partner [25]. While other authors have reported unidimensional aspects such as out of pocket costs [16, 17], this study revealed a number of other interesting economic aspects that have not been well described before in this urban and poor setting. Broadly speaking, economic influences could be grouped into direct and indirect factors. Direct economic influences include out-of-pocket costs such as transport and food [16]. For male partners who participated in the study, these direct economic costs did not seem to be a big factor in the decision to attend ANC. However, indirect costs or opportunity cost of not being at work seemed to have a far greater effect in the decision to attend ANC [25]. There were a couple of examples of how indirect economic costs impacted male partners’ decision. Firstly, spending more time at the hospital instead of engaging in an economic activity was a big factor [17, 25], compounded by informal self-employment or manual jobs with daily small income of often less than US$5 a day. Secondly, for those who were employed, they often feared remuneration deductions or even being dismissed for missing work. In order to remedy this, participants suggested that they would prefer to receive a formal invitation from the clinic which they could tender at their work place to obtain permission from their line managers for the ANC visit.

The study also found other barriers that are well documented in the literature. Structural problems associated with how the ANC service is offered which favours women as opposed to their male partners. It was observed in this study and other studies that while there are plenty of activities during the ANC visit, there is little male partner-centred focus in the curriculum [14]. This was particularly discouraging even to those male partners who had initially attended ANC in the first place who would later act as “negative” change agents by discouraging other men. A key facilitator to male partner ANC attendance mentioned by participants in this study was having a male friendly clinic [25]. Although other authors have suggested male protected time as a potential facilitator [17, 30], having a male friendly clinic encompassed more than protected time. Both male partners and ANC attending pregnant women agreed during the interviews that more male partners would feel comfortable attending a private, quick, smiling and flexible service that targets them and their pregnant partner. This finding was interesting as male partners did not express need for an entirely new place but simply a “culture” change within the same physical space of the primary clinic.

This study also revealed that cultural norms in Malawi view ANC as a woman’s space with male partners considered trespassers. Society beliefs that only women should attend ANC were prominent during FGDs and IDIs. Such an observation has been reported by a number of authors in different settings in Africa [3, 17]. Such cultural norms view ANC as a gendered setting with male partners exclusively assigned the role of the bread winner despite being responsible for the pregnancy while child bearing is strictly a domain for women.

As described by other researchers, extreme fear of couple testing for HIV among male partners was found to be a strong deterrent to ANC attendance [31]. In general, male partners believed that an ANC visit is synonymous to going for HIV testing. Although some male partners described it as protecting their fragile partner from emotional breakdown in a public place, narratives of infidelity almost explain this fear. Conversely, it was interesting to note that even some male partners suggested that testing negative together could cement their relationship with their partner. Such sentiments suggest that only a positive HIV test is feared by the male partners and not necessarily the testing. The fact that HIV testing at ANC is “batched” i.e. done in a group did not help matters with male partners feeling insecure about the lack of privacy and confidentiality particularly in the event that a positive test would be encountered.

Here three notable limitations of the study are given. Bangwe PHC had fewer male partners than Ndirande and Zingwangwa. First, given that male partners were the main focus of this analysis, findings may have limited scope to male partners of pregnant women from Bangwe PHC. Secondly, the selection of participants for the IDIs may have been too subjective given that the selection was after participants had participated in a FGD before. However, the selection was made in a quasi-random fashion albeit using names of focus group discussion participants in order to minimise the impact of this limitation. Finally, IDI participants were selected from FGD participants, therefore IDI responses may have been influenced by what respondents heard in FGDs.

## Conclusions

In summary, this study found that peer and economic influence were the strongest social barriers stopping male partners from attending antenatal care with their pregnant partners. Having a male friendly clinic - i.e. a room or rooms with a friendly service for male partners including flexible hours, private and confidential service and smiling personnel - was a novel facilitator suggested by participants.

## Supplementary Information

**Additional file 1.** focus group discussion guide.

**Additional file 2.** In-depth interview guide

## Data Availability

The final fully anonymised data from the study will be made publicly available through the LSHTM data repository (http://datacompass.lshtm.ac.uk/.

## Abbreviations

ANC: Antenatal care
COMREC: College of medicine research ethics committee
FGD: Focus group discussion
IDI: In-depth interviews
HIV: Human immunodeficiency virus
LSHTM: London school of hygiene and tropical medicine
PASTAL: Partner assisted self-testing and linkage
PEPFAR: President’s emergency plan for aids relief
PHC: Primary health centres
PLWH: People living with HIV
SSA: Sub-Saharan Africa
