# Supplementary material for: Socio-cultural and economic barriers, and facilitators influencing men’s involvement in antenatal care including HIV testing: a qualitative study from urban Blantyre, Malawi
Source: BMC Public Health. 2021 Jan 6;21:60. doi: 10.1186/s12889-020-10112-w (PMC7789341; doi:10.1186/s12889-020-10112-w)
Supplement: Supplementary file 1 — Additional file 1. focus group discussion guide. [file 12889_2020_10112_MOESM1_ESM.pdf]

PQ01 Focus Group Discussion Interview Guide: English & Chichewa
v0.3; 6th August 2015

Malawi-Liverpool-Wellcome Trust
Clinical Research Programme
P.O Box 30096, Chichiri, Blantyre 3, Malawi.
Tel. +265 1 876444 Fax +265 1 875774

Title: Developing contextually acceptable candidate interventions for increasing uptake of HIV testing and linkage into care or prevention for male partners of pregnant women in Antenatal clinics in Blantyre, Malawi: a cross-sectional qualitative study

Mutu: Kupeza njira zovomerezeka zochulukitsira chiwerengero cha abambo oyezetsa kachirombo ka HIV komanso kupita kolondira chithandizo choyeyenera pakati pa azibambo amene okondedwa awo amapita ku sikelo ya amayi oyembekezera mu mzinda wa Blantyre, Malawi.

General perceptions towards antenatal clinic (ANC) services

Zomwe anthu amaganiza zokhuzana ndi chithandizo chimene chimapezeka ku sikelo ya amayi oyembekezera.

1) What do people in your community say about men who attend antenatal clinic (ANC) services with their partners?

   Kodi anthu akudera kwanu amanena zotani zokhuzana ndi azibambo amene amaperekeza okondedwa awo ku sikelo ya amayi oyembekezera?

   Probe:

   a) What do community members feel about how ANC services are offered and organised?

      Kodi anthu amdera lanu akuona bwanji za mmene chithandizo chimene chimaperekedwa ku sikelo ya amayi oyembekezera?

   b) Which stories are positive and which ones are negative?

      Kodi ndi zonena zake ziti zimene zili zabwino komanso ndi zonena zake ziti zimene zisali zabwino?

   c) Which stories are much common?

      Kodi ndi zonena zake ziti zimene zimanenedwa kwambiri?

   d) What do you think makes the community members come up with these most common stories?

Kodi ndi chani chimene mukuganiza kuti chimapangitsa anthu kuti azinena zimene zimanenedwa kwambirizi?

2) What do people in your community say about men who test for HIV at ANC with their partners?

   Kodi anthu akudera kwanu amati chani zokhuzana ndi azibambo amene amayezetsa kachirombo ka HIV ku sikelo ya amayi oyembekezera limodzi ndi okondedwa awo?

   Probe:

   a) How do you feel about how ANC services are organised?

      Kodi chithandizo chimene chimaperekedwa ku sikelo ya amayi oyembekezera chimaperekedwa motani?

   b) Which stories are positive and which ones are negative?

      Kodi ndi zonena zake ziti zimene zili zabwino komanso ndi zonena zake ziti zimene zisali zabwino?

   c) Which stories are much common?

      Kodi ndi zonena zake ziti zimene zimanenedwa kwambiri?

   d) What do you think makes the community members come up with these most common stories?

      Kodi ndi chani chimene mukuganiza kuti chimapangitsa anthu kuti azinena zimene zimanenedwa kwambirizi?

Men's perceptions towards ANC services and HIV testing and counseling (HTC) at ANC

Zimene azibambo amaganiza zokhuzana ndi chithandizo chimene chimapezeka ku sikelo ya amayi oyembekezera komanso kuyezetsa ndi kulandira uphungu wa kachirombo ka HIV ku sikelo ya amayi oyembekezera

3) What do men say about attending ANC services with their partners?

   Kodi azibambo amanena zotani zokhudzana ndi kuperekeza okondedwa awo ku sikelo ya amayi oyembekezera?

   Probe:

   a) How do men feel about testing for HIV at ANC / how do men react towards testing for HIV at ANC with their partner?

Kodi azibambo amaona bwanji pa zoyezetsa kachirombo ka HIV limodzi ndi okondedwa awo ku sikelo ya amayi oyembekezera?

b) How do men feel about how HIV testing at ANC is organised?

Kodi azibambo amaona bwanji za mmene kuyezetsa kachirombo ka HIV ku sikelo ya amayi oyembekezera kumakhalira?

c) How do men feel about HIV care?

Kodi azibambo amaona bwanji za chisamaliro chimene chimaperekedwa kwa anthu opezeka ndi kachirombo ka HIV?

d) How do men feel about voluntary male medical circumcision?

Kodi azibambo amaziona bwanji za mdulidwe wa abambo wa kuchipatala?

e) What do you think prevents men who are escorting their pregnant women to ANC from testing for HIV?

Kodi mukuganiza kuti ndi chani chimene chimalepheretsa azibambo kuyezetsa kachirombo ka HIV pamene akuperekeza okondedwa awo ku sikelo ya amayi oyembekezera?

Perceptions about the acceptability of HIVST provided through ANC for men
Maganizo okhuzana ndi mmene anthu

4) What do you think about a clinic linked to ANC that offers HIV services for male partners of pregnant women only i.e. a male friendly clinic in terms of encouraging male partners to test and link?

   Kodi kukhala ndi kachipatala kopeleka thandizo lokhudzana ndi kachirombo ka HIV kwa azibambo amene ali ndi amayi oyembekezera cholumikizidwa ku sikelo ya amayi oyembekezera chingalimbikitse azimbambo kuyezetsa komanso kumapita kuchipatala akapezeka ndi kachirombo ka HIV?

5) In your opinion, would HIV self-testing (HIVST) provided through ANC be accepted amongst men with ANC attending partners?

   Malingana ndi mmene mukuonera, kodi mukugaiza kuti kuziyeza wekha kachirombo ka HIV kumene kumachitikira ku sikelo ya amayi oyembekezera kungakhale kovomerezeka pakati pa azibambo amene okondedwa awo amapita ku sikelo ya amayi oyembekezera?

6) In your opinion, would HIV self-testing (HIVST) provided through the woman on behalf of her partner (s) during ANC be accepted amongst men with ANC attending partners?

   Malingana ndi mmene mukuonera, kodi mukuganiza kuti kumpatsa mzimayi oyembekezera ku sikelo zipangizo zoziyezera wekha HIV kuti akampatse okondedwa wake polimbikitsa kuyezetsa pakati pa abambo kungakhale kovomerezeka?

7) What would be the community concerns to provide HIVST through ANC?

   Kodi mukuona kuti anthu amdera lanu angakhale ndi nkhawa yotani pa zoti kuziyeza wekha kachirombo ka HIV kuzichitikira ku sikelo ya amayi oyembekezera?

   Probe:
   a) Concerns or worries amongst men with ANC attending partners.

      Nkhawa kapena madandaulo amene azibambo amene okondedwa awo amapita ku sikelo atha kukhala nawo.

8) What should be done to make HIVST offered through ANC more acceptable to men with ANC attending partners?

   Kodi chikuyenera kuchitika ndi chani kuti kuziyekha wekha kachirombo ka HIV kumene kungamachitikire ku sikelo ya amayi oyembekezera kukhale kolandiridwa ndi azibambo amene okondedwa awo amapita ku sikelo ya amayi oyembekezera?

Perceptions about PASTAL interventions

9) How do you feel about the following approaches for encouraging male partners of pregnant women to test for HIV and link for appropriate services such as ART, counselling, condoms or voluntary male medical circumcision (VMMC)?

   Kodi maganizo anu ndi otani pa njira zili mu nsimu zolimbikitsa azibambo omwe okondedwa awo akupita ku sikelo kuti ayezetse kachirombo ka HIV komanso kuti ayambe kulandira chithandizo choyenerera monga mankhwala otalikitsa moyo, uphungu wa kachirombo ka HIV, makondomu kapena mdulidwe wa abambo wa kuchipatala?

   a) Standard of care - not introducing any change to HTC at ANC

      Kupitiriza kupereka chithandizo chimene chimaperekedwa nthawi zonse – osasintha china chilichonse pa ndondomeko yoyezera kachirombo ka HIV ndi kulandira uphungu ku sikelo ya amayi oyembekezera.

b) Providing HIV Self-test kits (ST) only e.g. provide two self-test kits to the woman to take home to discuss so that her partner self-tests with her or without her

Kupereka zipangizo zoziyezera wekha kachirombo ka HIV zokha basi. Mwachitsanzo, kuperekeza zipangizo zoziyezera wekha ziwiri kwa mzimayi kuti apititse kunyumba kuti akakambirane ndi wokondedwa wake kuti wokondedwa wake akathe kuziyeza yekha limodzi ndi iye kapena popanda iyeyo.

c) HIVST kits plus a low amount incentive i.e. an amount that would cover transport costs to the clinic.

Kupereka zipangizo zoziyezera wekha kachirombo ka HIV ndi kandalama kochepa kokwanira transport yokafikira kuchipatala.

d) HIVST kits plus a medium amount incentive i.e. an amount that would cover transport costs to the clinic plus some little compensation of time spent off economic activity.

Kupereka zipangizo zoziyezera wekha kachirombo ka HIV ndi ndalama yokwererapo pang’ono yokokwanira transport yokafikira kuchipatala komanso kandalama kena kochepa kongowathokoza chifukwa cha nthawi yimene aononga kapena asiya ntchito zawo zowapezera ndalama.

e) HIVST kits plus a high amount incentive ie an amount over and above transport costs to the clinic plus some little compensation of time spent off economic activity.

Kupereka zipangizo zoziyezera wekha kachirombo ka HIV ndi ndalama zopitilira transport yokafikira kuchipatala komanso kupitilira ndalama yongowathokoza chifukwa cha nthawi yimene aononga kapena asiya ntchito zawo zowapezera ndalama.

f) HIVST kits plus a lottery incentive i.e. 2 in 20 people will win a reasonably large sum through a raffle draw.

Kupereka zipangizo zoziyezera wekha kachirombo ka HIV komanso mphoto kudzera mmayere oti anthu awiri mwa anthu makumi awiri adzawine ndalama yochulukirapo.

10. How much in monetary value should the low, medium and high amount financial incentive interventions be?

Kodi ndalama zochuluka bwanji zimene zingaperekedwe pa njira zimene zatchulidwa m'mwambazi zolimbikitsira azibambo okhala ndi okondedwa oyembekezera kuti ayezetse kachirombo ka HIV (mwachitsanzo njira yokhala ndi ndalama yochepera; njira yokhala ndi ndalama yochuluka pan'gono, njira yokhala ndi ndalama yochuluka kwambiri)

11. Please rank these interventions on a scale of 1-5 to [e.g. 1=very unacceptable, 2=unacceptable, 3=not sure, 4=acceptable, 5=very acceptable].

    Chonde onetsani mmene mukuonera za kuvomerezeka kwa njira zimenezi pogwiritsa ntchito manambala. Mwachitsanzo, 1 kutanthauza yosavomerezeka kwambiri; 2 kutanthauza yosavomerezeka; 3 simukudziwa; 4 yovomerezeka; yovomerezeka kwambiri.

    Probe
    Which mode, cash or voucher, do you think would best work to encourage male partners to test and link?)
    Kodi ndi njira yiti, yopereka ndalama kapena vocha yogulira zinthu, imene yingalimbikitse kwambiri azibambo kuyezetsa komanso kupita kuchipatala akaona zotsatira zakuyezetsaku

    How long do you think it would take for the whole process to complete i.e. number of days for the male partner to test and link?

    Kodi pangatenge nthawi yochuluka bwanji kapena masiku angati kuchokera nthawi yimene mzibambo wazuyeza kufikira nthawi yopita kuchipatala kukapeza thandizo?

Introducing and implementing PASTAL interventions

12. Do you think ANC attending women would be able to introduce HIVST to their male partners? What do you think would be the possible consequences to these women when they introduce HIVST to their male partners?

    Kodi mukuganiza kuti amayi amene amapita ku sikelo ya amayi oyembekezera angathe kuwafotokozera okondedwa awo zokhuzana kuziyeza kachirombo ka HIV? Mukuganiza kuti chimene chingachitikire amayi amenewawa ndi chani pamene akuwafotokozera okondedwa awo za kuziyeza wekha kachirombo ka HIV?

    Probe:
    a) Issues of coercion/intimate partner violence

    Funsani zokhuzana ndi kuwumirizana kapena nkhanza zochitirana mchikondi

13. How could each of the following interventions be implemented at ANC to make it more acceptable and preferred by men with ANC attending partners?

    Kodi njira zotsatirazi zingayendetsedwe motani kuti azibambo amene okondedwa awo amapita ku sikelo ya amayi oyembekezera athe kuzivomera ndi kuzikonda kwambiri?

    a) Providing HIV Self-test kits (ST) only e.g. provide two self-test kits to the woman to take home to discuss so that her partner self-tests with her or without her

       Kupereka zipangizo zoziyezera wekha kachirombo ka HIV zokha basi. Mwachitsanzo, kupereka zipangizo zoziyezera wekha ziwiri kwa mzimayi kuti apititse kunyumba kuti akakambirane ndi wokondedwa wake kuti wokondedwa wake akathe kuziyeza yekha limodzi ndi iye kapena popanda iyeyo.

    b) HIVST kits plus a low amount incentive i.e. an amount that would cover transport costs to the clinic.

       Kupereka zipangizo zoziyezera wekha kachirombo ka HIV ndi kandalama kochepa kokwanira transport yokafikira kuchipatala.

    c) HIVST kits plus a medium amount incentive i.e. an amount that would cover transport costs to the clinic plus some little compensation of time spent off economic activity.

       Kupereka zipangizo zoziyezera wekha kachirombo ka HIV ndi ndalama yokwererapo pang'ono yokokwanira transport yokafikira kuchipatala komanso kandalama kena kochepa kongowathokoza chifukwa cha nthawi yimene aononga kapena asiya ntchito zawo zowapezera ndalama.

    d) HIVST kits plus a high amount incentive ie an amount over and above transport costs to the clinic plus some little compensation of time spent off economic activity.

       Kupereka zipangizo zoziyezera wekha kachirombo ka HIV ndi ndalama zopitilira transport yokafikira kuchipatala komanso kupitilira ndalama yongowathokoza chifukwa cha nthawi yimene aononga kapena asiya ntchito zawo zowapezera ndalama.

e) HIVST kits plus a lottery incentive i.e. 2 in 20 people will win a large sum through a raffle draw.

Kupereka zipangizo zoziyezera wekha kachirombo ka HIV komanso mphoto kudzera mmayere oti anthu awiri mwa anthu makumi awiri adzawine ndalama zambiri.

14. What would be other approaches that could be used to increase uptake of HIV testing and linkage into care or prevention for male partners of ANC attending women?

Kodi ndi njira zina ziti zimene zingathe kugwiritsidwa ntchito kuti azibambo ambiri amene okondedwa awo amapita ku sikelo ya amayi oyembekezera azitha kuyezetsa kachirombo ka HIV komanso kuyambitsidwa kulandira chisamaliro?
