# Supplementary material for: Socio-cultural and economic barriers, and facilitators influencing men’s involvement in antenatal care including HIV testing: a qualitative study from urban Blantyre, Malawi
Source: BMC Public Health. 2021 Jan 6;21:60. doi: 10.1186/s12889-020-10112-w (PMC7789341; doi:10.1186/s12889-020-10112-w)
Supplement: Supplementary file 2 — Additional file 2. In-depth interview guide [file 12889_2020_10112_MOESM2_ESM.pdf]

## PQ02 In-depth Interview Guide: English & Chichewa
**v0.3; 16th January 2016**

**Malawi-Liverpool-Wellcome Trust**
**Clinical Research Programme**
P.O Box 30096, Chichiri, Blantyre 3, Malawi.
Tel. +265 1 876444 Fax +265 1 875774

**Title:** Developing contextually acceptable candidate interventions for increasing uptake of HIV testing and linkage into care or prevention for male partners of pregnant women in Antenatal clinics in Blantyre, Malawi: a cross-sectional qualitative study

**Mutu:** Kupeza njira zovomerezeka zochulukitsira chiwerengero cha abambo oyezetsa kachirombo ka HIV komanso kupita kolondira chithandizo choyeyenera pakati pa azibambo amene okondedwa awo amapita ku sikelo ya amayi oyembekezera mu mzinda wa Blantyre, Malawi.

### General perceptions towards antenatal clinic (ANC) services

### Zomwe anthu amaganiza zokhuzana ndi chithandizo chimene chimapezeka ku sikelo ya amayi oyembekezera.

1) What do you feel about you (if male respondent) or your male partner (if female respondent) attending antenatal clinic (ANC) services?

   Kodi inu mumaganiza zotani pankhani yokhuzana ndi inuyo (if male respondent) kapena wachikondi wanu (if female respondents) kukapezeka ku sikelo ya amayi oyembekezera?

   **Probe:**

   a) What do you feel about how ANC services are offered and organised?

      Kodi inu maganizo anu ndi otani okhuzana ndi mmene chithandizo chimaperekedwera ku sikelo ya amayi oyembekezera?

   b) What do you to feel that way about services offered at ANC?

      Kodi ndi chani chimene chimabweresa maganizo amenewa pa nkhani yokhuza thandizo limene limaperekedwa ku sikelo ya amayi oyembekezera?

2) What do you feel about men who test for HIV at ANC with their partners?

   Kodi mumaganiza zotani zokhuzana ndi azibambo amene amayezetsa kachirombo ka HIV ku sikelo ya amayi oyembekezera limodzi ndi okondedwa awo?

   **Probe:**

a) How do you feel about how HIV testing services are organised at ANC clinic?

   Kodi maganizo anu ndi otani pankhani yokhuzana ndi kuyezesa kachirombo ka HIV ku sikelo ya amayi oyembekezera?

**Men's perceptions towards ANC services and HIV testing and counseling (HTC) at ANC**

Zimene azibambo amaganiza zokhuzana ndi chithandizo chimene chimapezeka ku sikelo ya amayi oyembekezera komanso kuyezetsa ndi kulandira uphungu wa kachirombo ka HIV ku sikelo ya amayi oyembekezera

3) What do you feel (for male respondent) / your partner (for female respondents) feel about attending ANC services with their partners?

   Kodi inu (for male respondent) / okondedwa wanu (for female respondents) mumaganiza/amaganiza zotani zokhudzana ndi kupitak ku sikelo ya amayi oyembekezera pamodzi ndi wachokondi?

   **Probe:**

   a) How do you (for male respondent) / your partner (for female respondents) feel about testing for HIV at ANC / how do you (for male respondent) / your male partner (for female respondents) react towards testing for HIV at ANC with their partner?

      Kodi inu (for male respondent) / okondedwa wanu (for female respondents) mumaona/amaona bwanji pa zoyezetsa kachirombo ka HIV limodzi ndi okondedwa awo ku sikelo ya amayi oyembekezera?

   b) How do you (for male respondent) / your partner (for female respondents) feel about how HIV testing at ANC is organised?

      Kodi inu (for male respondent) / okondedwa wanu (for female respondents) mumaona/amaona bwanji za mmene kuyezetsa kachirombo ka HIV ku sikelo ya amayi oyembekezera kumakhalira?

   c) How do you (for male respondent) / your partner (for female respondents) feel about HIV care?

      Kodi inu (for male respondent) / okondedwa wanu (for female respondents) mumaona/amaona bwanji za chisamaliro chimene chimaperekedwa kwa anthu opezeka ndi kachirombo ka HIV?

   d) How do you (for male respondent) / your partner (for female respondents) feel about voluntary male medical circumcision?

      Kodi inu (for male respondent) / okondedwa wanu (for female respondents) mumaona/amaona bwanji za mdulidwe wa abambo wa kuchipatala?

e) What do you think prevents you (for male respondent) / your partner (for female respondents) who are escorting their pregnant women to ANC from testing for HIV?

Kodi mukuganiza kuti ndi chani chimene chimalepheretsa inu (for male respondent) / okondedwa wanu (for female respondents) mumaona/amaona kuyezetsa kachirombo ka HIV pamene akuperekeza okondedwa awo ku sikelo ya amayi oyembekezera?

**Perceptions about the acceptability of HIVST provided through ANC for men**
**Maganizo okhuzana ndi kubvomerezeka kwa ndondomeko yoziyeza wekha kachirombo ka HIV ku sikelo ya amayi oyembekezera**

4) What do you think about a clinic linked to ANC that offers HIV services for male partners of pregnant women only i.e. a male friendly clinic in terms of encouraging male partners to test and link?

   Kodi kukhala ndi kachipatala kopeleka thandizo lokhudzana ndi kachirombo ka HIV kwa azibambo amene ali ndi amayi oyembekezera cholumikizidwa ku sikelo ya amayi oyembekezera chingalimbikitse azimbambo kuyezetsa komanso kumapita kuchipatala akapezeka ndi kachirombo ka HIV?

5) In your opinion, would HIV self-testing (HIVST) provided through ANC be accepted by you / your male partner?

   Malingana ndi mmene mukuonera, kodi mukugaiza kuti kuziyeza wekha kachirombo ka HIV kumene kumachitikira ku sikelo ya amayi oyembekezera kungakhale kovomerezeka kwa inuyo (for male respondent) / Okondedwa wanu (for female respondents)?

6) In your opinion, would HIV self-testing (HIVST) provided through the woman on behalf of her partner (s) during ANC be accepted amongst men with ANC attending partners?

   Mmene mukuonera, kodi mukuganiza kuti kumpatsa mzimayi oyembekezera zipangizo zoziyezera wekha HIV akapitata kusikelo kuti akampatse okondedwa wake polimbikitsa kuyezetsa pakati pa abambo kungakhale kovomerezeka?

7) What would be your concerns to provide HIVST through ANC?

   Kodi muli ndi nkhawa yotani pankhani yokhala ndi ndondomeko yoziyeza wekha kachirombo ka HIV kuzezera ku sikelo ya amayi oyembekezera?

   **Probe:**

   a) Concerns or worries amongst men with ANC attending partners and ANC attending women

Nkhawa kapena madandaulo amene azibambo amene okondedwa awo amapita ku sikelo atha kukhala nawo komanso nkhawa za azimayi amene amapita kusikelo ya amayi oyembekezera.

8) What should be done to make HIVST offered through ANC more acceptable to men with ANC attending partners?

   Kodi ndi chani chimene chikuyenera kuchitika kuti kuziyekha wekha kachirombo ka HIV kumene kungamachitikire ku sikelo ya amayi oyembekezera kukhale kobvomenerezeka pakati pa azibambo amene okondedwa awo amapita ku sikelo ya amayi oyembekezera?

**Perceptions about PASTAL interventions**

9) How do you feel about the following approaches for encouraging male partners of pregnant women to test for HIV and link for appropriate services such as ART, counselling, condoms or voluntary male medical circumcision (VMMC)?

   Kodi maganizo anu ndi otani pa njira zili m'munsimu zolimbikitsa azibambo omwe okondedwa awo akupita ku sikelo kuti ayezetse kachirombo ka HIV komanso kuti ayambe kulandira chithandizo choyenerera monga mankhwala otalikitsa moyo, uphungu wa kachirombo ka HIV, makondomu kapena mdulidwe wa abambo wa kuchipatala?

   a) Standard of care - not introducing any change to HTC at ANC

      Kupitiriza kupereka chithandizo chimene chimaperekedwa nthawi zonse – osasintha china chilichonse pa ndondomeko yoyezera kachirombo ka HIV ndi kulandira uphungu ku sikelo ya amayi oyembekezera.

   b) Providing HIV Self-test kits (ST) only e.g. provide two self-test kits to the woman to take home to discuss so that her partner self-tests with her or without her

      Kupereka zipangizo zoziyezera wekha kachirombo ka HIV zokha basi. Mwachitsanzo, kupereka zipangizo zoziyezera wekha ziwiri kwa mzimayi kuti apititse kunyumba kuti akakambirane ndi wokondedwa wake kuti wokondedwa wake akathe kuziyeza yekha limodzi ndi mkazi wake kapena popanda mkazi.

   c) HIVST kits plus a low amount incentive i.e. an amount that would cover transport costs to the clinic.

      Kupereka zipangizo zoziyezera wekha kachirombo ka HIV ndi kandalama kochepa kokwanira transport yokafikira kuchipatala.

d) HIVST kits plus a high amount incentive ie an amount over and above transport costs to the clinic plus some little compensation of time spent off economic activity.

   Kupereka zipangizo zoziyezera wekha kachirombo ka HIV ndi ndalama zopitilira transport yokafikira kuchipatala komanso kupitilira ndalama yongowathokoza chifukwa cha nthawi yimene aononga kapena asiya ntchito zawo zopezera ndalama.

e) HIVST kits plus a lottery incentive i.e. 2 in 20 people will win a reasonably large sum through a raffle draw.

   Kupereka zipangizo zoziyezera wekha kachirombo ka HIV komanso mphoto kudzera mmayere oti anthu awiri mwa anthu makumi awiri adzawine ndalama yochulukirapo.

f) HIVST kits followed by phone call reminder
   Kupereka zipangizo zoziyezera wekha kachirombo ka HIV komanso kuti abambowo tidziwayimbira foni ndikukambirana nawo zoziyeza wekha.

   **Probe:**

   i) Should the phone be made before the woman talks to her partner?
      Kodi foniyi idzyimbidwa nthawi yanji poyamba? Mzimayi asanafikitse nkhaniyi kwa a bamboo?

   ii) How many times should the reminder be provided?
      Kodi abambowa tidziwayimbira kangati kuphatikiza kuyimbiridwa koyamba mpaka kumaliza zonse?

10. How much in monetary value should the low, medium and high amount financial incentive interventions be?

    Kodi ndi ndalama zochuluka bwanji zimene zingaperekedwe pa njira zimene zatchulidwa m'mwambazi zolimbikitsira azibambo okhala ndi okondedwa oyembekezera kuti ayezetse kachirombo ka HIV (mwachitsanzo njira yokhala ndi ndalama yochepera; njira yokhala ndi ndalama yochuluka pan'gono, njira yokhala ndi ndalama yochuluka kwambiri)

11. Which mode, cash or voucher, do you think would best work to encourage male partners to test and link?)
    Kodi ndi njira yiti, yopereka ndalama kapena vocha yogulira zinthu, imene yingalimbikitse kwambiri azibambo kuyezetsa komanso kupita kuchipatala akaona zotsatira zakuyezetsaku

**Introducing and implementing PASTAL interventions**

12. Do you think ANC attending women would be able to introduce HIVST to their male partners? What do you think would be the possible consequences to these women when they introduce HIVST to their male partners?

    Kodi mukuganiza kuti amayi amene amapita ku sikelo ya amayi oyembekezera angathe kubweretsa kwa okondedwa awo njira yoziyeza kachirombo ka HIV? Mukuganiza kuti chimene chingachitikire amayi amenewawa ndi chani pamene abweretsa zipangizo zoziyezera wekha kachirombo ka HIV kwa okondedwa awo?

    **Probe:**

    a) Issues of coercion/intimate partner violence

    Funsani zokhuzana ndi kuwumirizana kapena nkhanza zochitirana mchikondi

13. How could each of the following interventions be implemented at ANC to make it more acceptable and preferred by men with ANC attending partners?

    Kodi njira zotsatirazi zingakhazikitsidwe motani kuti azibambo amene okondedwa awo amapita ku sikelo ya amayi oyembekezera athe kuzivomera ndi kuzikonda kwambiri?

    a) Providing HIV Self-test kits (ST) only e.g. provide two self-test kits to the woman to take home to discuss so that her partner self-tests with her or without her

       Kupereka zipangizo zoziyezera wekha kachirombo ka HIV zokha basi. Mwachitsanzo, kupereka zipangizo zoziyezera wekha ziwiri kwa mzimayi kuti apititse kunyumba kuti akakambirane ndi wokondedwa wake kuti wokondedwa wake akathe kuziyeza yekha limodzi ndi mkazi wake kapena popanda mkazi.

    b) HIVST kits plus a low amount incentive i.e. an amount that would cover transport costs to the clinic.

       Kupereka zipangizo zoziyezera wekha kachirombo ka HIV ndi kandalama kochepa kokwanira transport yokafikira kuchipatala.

    c) HIVST kits plus a high amount incentive ie an amount over and above transport costs to the clinic plus some little compensation of time spent off economic activity.

       Kupereka zipangizo zoziyezera wekha kachirombo ka HIV ndi ndalama zopitilira transport yokafikira kuchipatala komanso kupitilira ndalama yongowathokoza chifukwa cha nthawi yimene awononga kapena asiya ntchito zawo zopezera ndalama.

d) HIVST kits plus a lottery incentive i.e. 2 in 20 people will win a large sum through a raffle draw.

Kupereka zipangizo zoziyezera wekha kachirombo ka HIV komanso mphoto kudzera mmayere oti anthu awiri mwa anthu makumi awiri adzawine ndalama zambiri.

g) HIVST kits followed by phone call reminder
Kupereka zipangizo zoziyezera wekha kachirombo ka HIV komanso kuti abambowo tidziwayimbira foni ndikukambirana nawo zoziyeza wekha.

14. What would be other approaches that could be used to increase uptake of HIV testing and linkage into care or prevention for male partners of ANC attending women?

Kodi ndi njira zina ziti zimene zingathe kugwiritsidwa ntchito kuti azibambo ambiri amene okondedwa awo amapita ku sikelo ya amayi oyembekezera azitha kuyezetsa kachirombo ka HIV komanso kuyambitsidwa kulandira chisamaliro?
